# First clinical implementation of insertion force measurement in cochlear implantation surgery

**DOI:** 10.3389/fneur.2024.1400455

**Published:** 2024-04-22

**Authors:** Thomas S. Rau, Georg Böttcher-Rebmann, Viktor Schell, Jakob Cramer, Eralp Artukarslan, Claas Baier, Thomas Lenarz, Rolf Salcher

**Affiliations:** ^1^Department of Otolaryngology and Cluster of Excellence EXC 2177/1 “Hearing4all”, Hannover Medical School, Hannover, Germany; ^2^Institute for Medical Microbiology and Hospital Epidemiology, Hannover Medical School, Hannover, Germany

**Keywords:** cochlear implant, insertion forces, insertion trauma, hearing preservation, force measurement, real-time monitoring, force monitoring

## Abstract

**Purpose:**

The significance of atraumatic electrode array (EA) insertion in cochlear implant (CI) surgery is widely acknowledged, with consensus that forces due to EA insertion are directly correlated with insertion trauma. Unfortunately, the manual perception of these forces through haptic feedback is inherently limited, and techniques for *in vivo* force measurements to monitor the insertion are not yet available. Addressing this gap, we developed of a force-sensitive insertion tool capable of capturing real-time insertion forces during standard CI surgery.

**Methods:**

This paper describes the tool and its pioneering application in a clinical setting and reports initial findings from an ongoing clinical study. Data and experiences from five patients have been evaluated so far, including force profiles of four patients.

**Results:**

The initial intraoperative experiences are promising, with successful integration into the conventional workflow. Feasibility of *in vivo* insertion force measurement and practicability of the tool’s intraoperative use could be demonstrated. The recorded *in vivo* insertion forces show the expected rise with increasing insertion depth. Forces at the end of insertion range from 17.2 mN to 43.6 mN, while maximal peak forces were observed in the range from 44.8 mN to 102.4 mN.

**Conclusion:**

We hypothesize that this novel method holds the potential to assist surgeons in monitoring the insertion forces and, thus, minimizing insertion trauma and ensuring better preservation of residual hearing. Future data recording with this tool can form the basis of ongoing research into the causes of insertion trauma, paving the way for new and improved prevention strategies.

## 1 Introduction

The preservation of residual hearing constitutes an important objective in the field of cochlear implant (CI) surgery. This is particularly true given the continuing expansion of inclusion criteria for this surgical procedure to encompass patients with increasing amounts of residual hearing (1–3). Atraumatic electrode array insertion, in turn, is the cornerstone of any strategy aimed at preserving residual hearing and intracochlear structures. Over the past two decades, research has been driven by the hypothesis that forces exerted during the placement of CI electrode arrays (EAs) are directly correlated with insertion trauma (4–7). If this hypothesis holds true, insertion forces could potentially serve as an indicator for both the occurrence and the extent of intracochlear damage. Although the importance of atraumatic EA insertion has been widely acknowledged, the manual perception of these forces through haptic feedback is very limited (5) as even small forces down to 42 mN are reported to cause intracochlear trauma (8) and, therefore, also intraoperative force measurements in humans have remained an unsolved challenge (9, 10).

The absence of such measurements in clinical practice severely limits the investigation and quantification of the impact of insertion forces on hearing outcome from a scientific perspective. The available evidence from preclinical research suggests, that aside from the mechanical characteristics of the electrode array [including, for example, stiffness and surface friction properties; (11–13)], patient-specific anatomical parameters [such as cochlear size and curvature; (14, 15)] also procedural factors [e.g., insertion trajectory, speed, depth; (16, 17)] cause individual variations. Building upon this, a deeper understanding of this relationship may pave the way for the development of novel strategies that empower clinicians to identify and mitigate the risk of intracochlear trauma in real time. Consequently, the ability to measure insertion forces intraoperatively also seems to hold high clinical relevance.

The absence of *in vivo* insertion force data motivated our research, leading to the development of an insertion tool capable of measuring insertion forces in real time during standard cochlear implant surgery, the “Forception Tool.” It already underwent extensive testing in laboratory settings, both in artificial cochlear models (18) and in human temporal bone specimens (19).

These experiments confirmed the quality and feasibility of the measurement. However, due to expected differences in tissue properties between *in vivo* and postmortem cochleae as well as inevitable limitations in simulating the entire surgical workflow using TB specimens, investigating the principle of insertion force measurement *in vivo* was the next logical and essential step toward the clinical use of this new technology. This paper describes the pioneering application of the tool in the clinical setting. Additionally, we present initial findings from this ongoing clinical study and will discuss the potential value of these insights for future clinical practice and the continued advancement of insertion techniques.

## 2 Materials and methods

### 2.1 Description of the force sensing insertion tool

**Figure 1:**
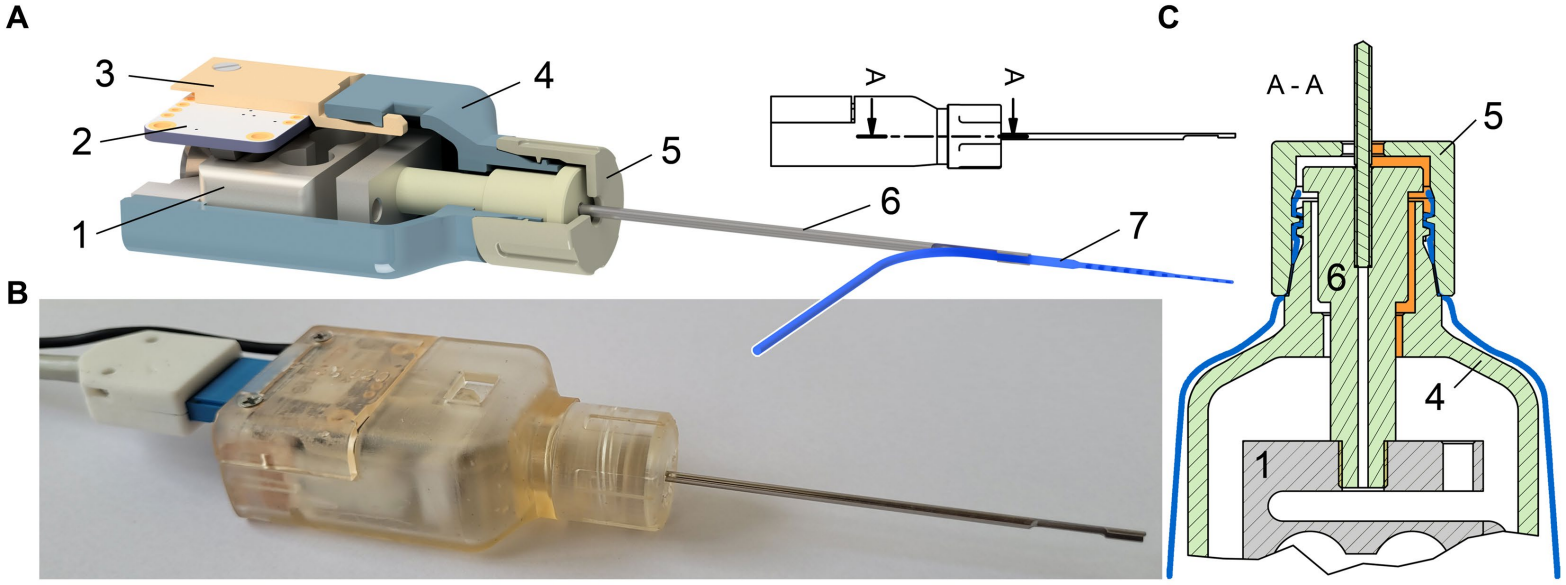
**(A)** Technical drawing of the Forception Tool. The section view of the housing allows the view inside the tool showing the force sensor (1), IMU (2), back part of housing (3), front part of housing (4), screw-on cap (5), and the electrode holder (6) with the grasped electrode array (7). **(B)** Photograph of the Forception Tool without sterile drape. **(C)** Cross section of the front part of the tool showing the labyrinth seal highlighted in orange. Green parts are delivered sterile into the OR while the gray force sensor is one of the non-sterile components inside the housing. The sterile drape is drawn in blue.

The tool was designed to replace the conventional forceps or tweezers normally used by the surgeon to hold the EA. Instead, the EA is clamped into a U-shaped holder made of stainless steel that extends from the front section of the tool. The clamping zone with an inner diameter of 1.2 mm was adapted to FLEX series EAs (MED-EL, Innsbruck, Austria), ensuring secure fixation without harming the EA. The other end of the electrode holder is made of PEEK (polyether ether ketone) to ensure electrical insulation between the device and the patient. This end can be screwed into a force sensor (KD24s, ME-Meßsysteme GmbH, Hennigsdorf, Germany) located inside the tool housing. Therefore, forces generated between the implant and the surrounding tissue in the direction of the main axis of the tool are transmitted through the electrode holder via the front opening of the insertion tool to the internal 1-dimensional force sensor (Figure 1).

Since changes in the tool’s orientation during manual use cause fluctuations in the measured force due to gravitational effects, the tool also contains an inertial measurement unit (IMU, BNO055, Bosch Sensortec GmbH, Reutlingen, Germany). This additional sensor records the tool’s orientation within the gravitational field, which, in turn, allows for the compensation of gravity-related artifacts in the force signal, enabling isolation of the desired insertion forces. Details about the gravity compensation can be found in (18).

The electrode holder is designed to be oriented toward the chorda-facial angle, which is why there are both left and right versions of the holder included in the surgical basket. This design choice is based on the concept that, after full insertion, the EA can be secured within a small bone groove created in the posterior tympantomy for electrode array fixation (20, 21). Using a second instrument, such as a blunt needle, the EA can be moved directly from the holder into the opposing bone groove, thereby minimizing intracochlear movements after reaching the desired insertion depth.

**Figure 2:**
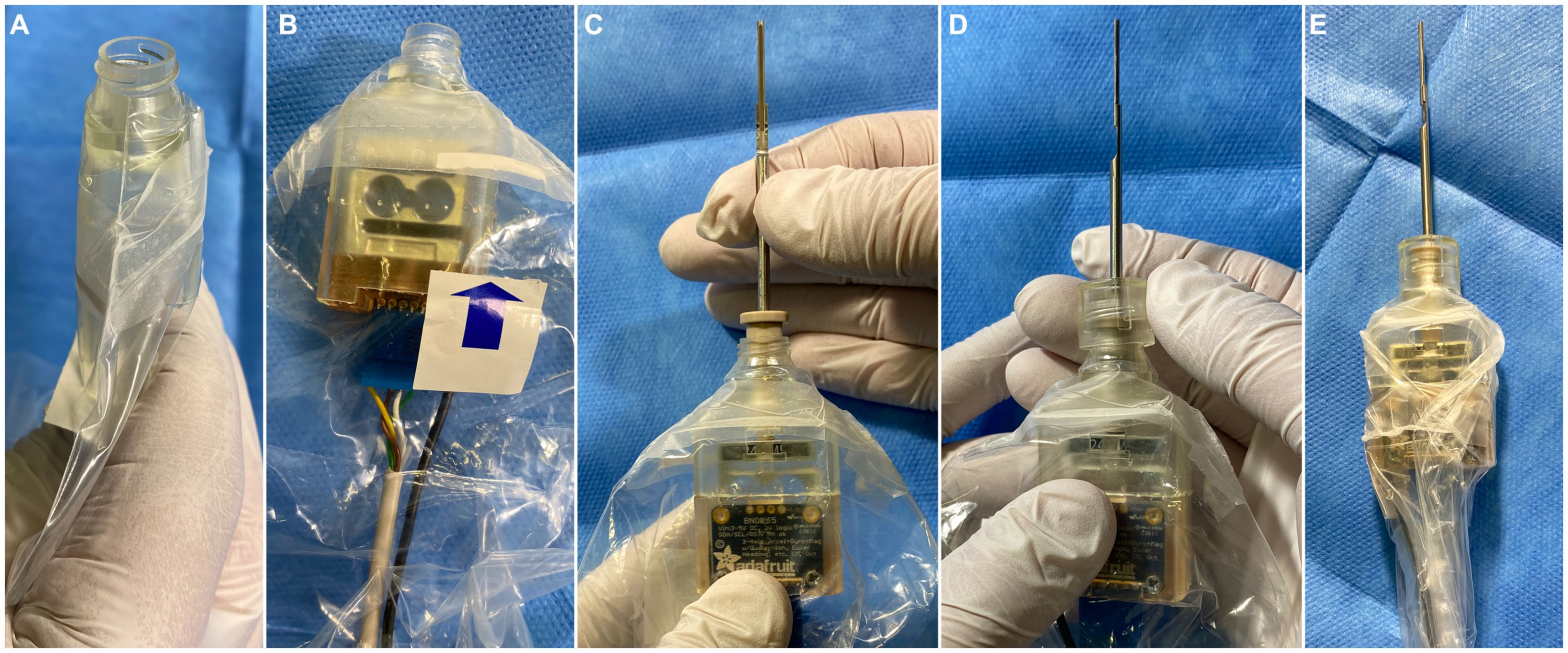
Sterile assembly of the tool. **(A)** Insertion of the sterile front of the housing into the drape, ensuring a tight fit at the opening of the drape. **(B)** Insertion of the non-sterile sensor unit, which is connected to the front part of the housing using a snap mechanism. **(C)** Insertion of the sterile EA holder from the front involves carefully screwing it into the force sensor inside the housing. **(D)** Mounting of the screw-on cap to fixate the sterile drape. **(E)** Tightening and taping of the drape around the tool using adhesive tape to prevent obstructions of the line-of-sight.

The 3D-printed housing (Surgical Guide, Formlabs Inc., Somerville, MA, US) is intentionally designed with a narrow side so that the line-of-sight past the tool remains unobstructed. In addition, the housing enables the safe division between sterile and non-sterile parts (e.g., electronic components). The solution we devised to keep these parts separate combines the envelopment by a sterile drape with small clearings. The critical aspect lies in the necessary opening within the tool’s housing, which necessitates a corresponding opening in the sterile barrier. Without such an opening the measurement of the very small insertion forces would be compromised as even a thin foil could dampen the transmission and prevent accurate force measurements. Owing to the presence of this hole, it is crucial to ensure that no liquids (such as blood, irrigation fluids, or rinsing solutions) can reach non-sterile parts. This is necessary both to protect the electronic components and to prevent the possibility of fluids returning to the surgical field after contact with non-sterile surfaces inside the tool. Hence, both the outer shape of the PEEK coupler and housing aperture are designed to form a labyrinth seal. This design choice was inspired from similar seals used in axle bearings and results in a convoluted and extended path preventing a direct entry or exit of liquids. To complete the labyrinth seal, a screw-on cap is used, serving the additional purpose of securing the sterile drape in place. The electrode holders and a screw-on cap are sterilely supplied to the operating room. Unlike in the previously presented versions, the front part of the housing is sterilized as well. This eliminates any risk of contact with non-sterile surfaces by the operating room staff, even during assembly (Figure 2).

The force sensor of the tool is connected to a laptop outside the surgical field via an amplifier (GSV-8, ME-Meßsysteme GmbH), while the IMU is connected using an Arduino Uno R3 microcontroller. A custom software was developed to record sensor data at up to 100 Hz, synchronize different data sources, perform gravity compensation, and visualize the measurements. It was created using the QT 5.12.9 framework (The QT Company, Espoo, Finland) and implemented in C++ (22). In addition, the software allows the simultaneous recording of up to two videos synchronized with the data, of which one was used to capture the live images from the digital surgical microscope (ARRISCOPE, Munich Surgical Imaging GmbH, Munich, Germany). This enables correlation of specific phenomena in the force data (e.g., peaks) with specific surgical events visible in the video.

### 2.2 Clinical trial

A first-in-human study was planned in order to prove the feasibility of intraoperative insertion force measurements, assess the integrability into the surgical workflow, and acquire initial *in vivo* force data. The latter aimed to glean preliminary insights into human *in vivo* insertion force profiles, including their typical trajectory, recurrent patterns, and the overall magnitude of insertion forces in living human subjects. The clinical study was planned in accordance with the “Professional Code for Physicians in Germany” and underwent review by the local institutional review board (10296_BO_S_2022). All patients gave written informed consent after they have received detailed information about the study, its purpose and potential risks. For safety reasons, only patients who do not have any usable residual hearing were considered. In addition, the insertion forces were recorded solely for scientific purposes and were not visualized to the surgeon during the procedure to prevent any potential interference with the surgeon’s behavior and decision-making. Real-time visualization of the measured forces is subject to a following clinical study.

In all patients, the conventional transmastoid posterior tympanotomy approach was used, and the EA was introduced through the round window. Two cases involved revision surgery; while in the remaining cases all steps of the standard preparation protocol were conducted, including: mastoidectomy, preparation of the implant bed for the receiver/stimulator including a tunnel into the mastoid cavity, posterior tympanotomy, creation of a bone groove in the inferior corner of the facial recess, and exposure of the round window membrane. Simultaneously, the tool was prepared for intraoperative use (Figure 2). Prior to encasing the tool in the sterile drape, the sensors were calibrated outside the surgical field using test weights for the force sensor and a specific movement routine for the IMU. Later, just before the start of the insertion, the sensors were tared again to compensate for intermediate drift.

**Figure 3:**
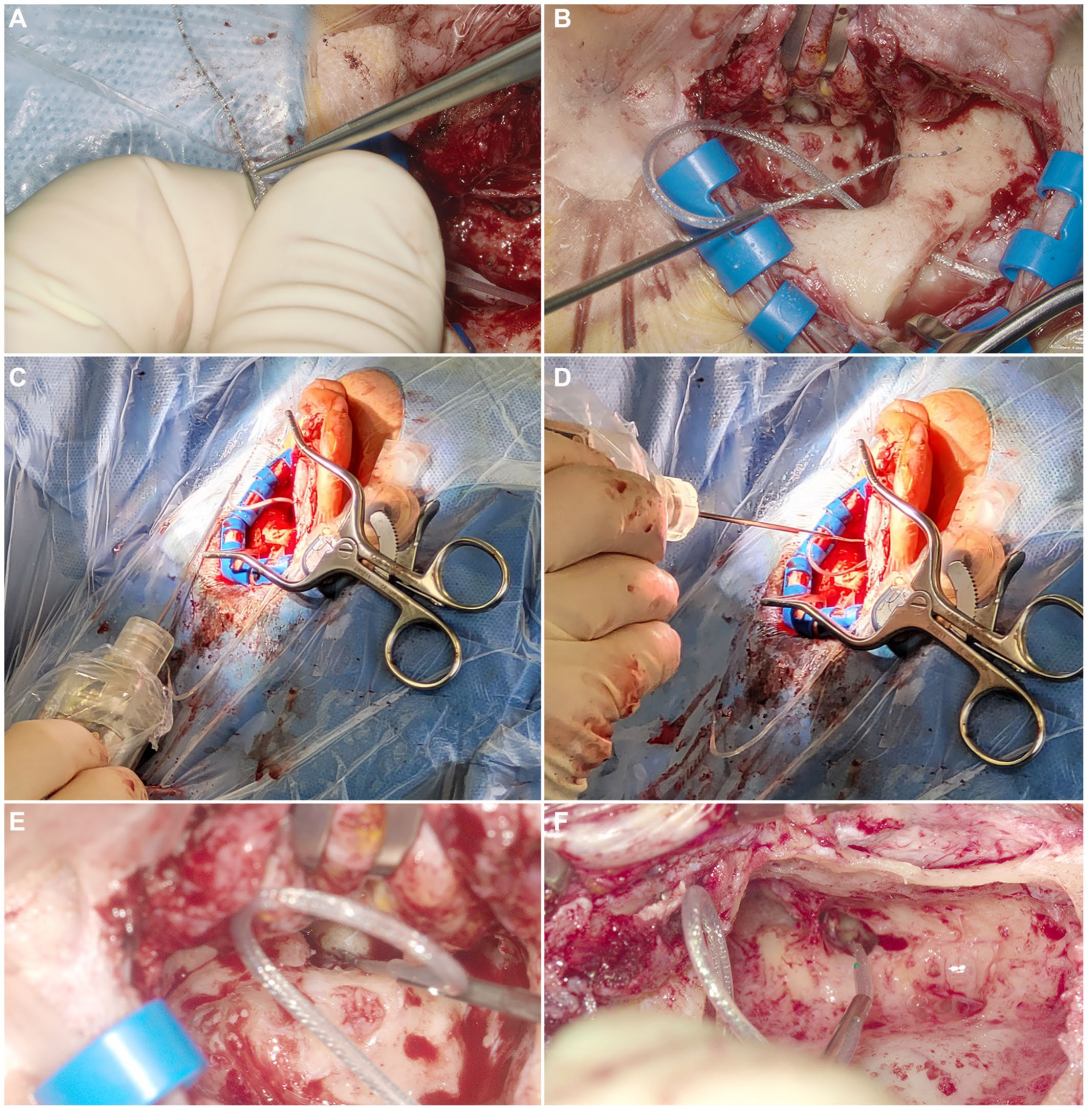
**(A)** Clamping of the EA into the U-shaped electrode holder. **(B)** Grasped EA. **(C)** Holding the EA with the Forception Tool right before the start of the insertion. **(D)** During electrode insertion. **(E,F)** View through the OR microscope during insertion in two different patients.

After placing the implant housing in its predrilled bed and puncturing of the round window membrane, the EA was clamped into the electrode holder (Figure 3A, Flex28 in all cases). The insertion was performed manually as carefully and slowly as possible avoiding contact between the electrode holder and the bony walls of the mastoid cavity and the facial recess (Figure 3). After the insertion, the EA was released from the tool and the surgery was finished according to the common clinical routine.

### 2.3 Data analysis

Surgeons were asked to provide feedback on their experience with the tool and note anything unexpected encountered during its use. They were specifically asked to highlight problems or difficulties observed, particularly in comparison to the conventional workflow. This included the following aspects of the tools usability and feasibility of the measuring method: (1) Whether the insertion was subjectively rated as successful (i.e., achieving the desired insertion depth); (2) Whether the EA was held securely and reliably by the tool during the entire insertion process; (3) Whether the tool notably impacted the visibility of the electrode array in the mastoid cavity and subsequently affected the visual monitoring of the insertion process.

The recorded forces were visually analyzed for any notable peaks or increased noise indicating potential bone contact of the tool tip and therefore interfering force generation outside the cochlea. The synchronous video footage was used to verify these suspected cases and remove these sections from the force data before subsequent evaluation, e.g., calculating average and maximum forces. Graphs and data analysis were performed in MATLAB 2023a (The Mathworks, Inc., Natick, MA, US).

## 3 Results

In May 2023, to the best of our knowledge, insertion forces were recorded intraoperatively for the first time worldwide. Data and experiences from five patients have been evaluated so far and are presented in the following.

All insertions were conducted by two highly experienced senior surgeons from our department. They reported no significant changes in the surgical workflow due to the use of the tool. The secure fixation of the EA in the electrode holder during the whole insertion rendered it equally maneuverable as with conventional instruments. Full insertion was possible as planned, visibility of the surgical field was not appreciably impaired, and no obstruction of the line of sight was reported by the surgeons. After insertion, the EA was released from the electrode holder and positioned into the groove in the chorda-tympani-angle for its final position.

Sterile encasing of the tool was performed by the surgical nurse in parallel to previous steps of the surgery. Therefore, only the fixation of the EA in the electrode holder minimally prolonged the surgery and was experienced by the surgeons as the most challenging step when using the tool.

In all cases except one, the recording of raw data as well as the gravity compensated forces and the video from the surgical microscope was successful. In the second patient, a lost connection to the IMU led to an outage of the gravity compensation, leaving the measured forces affected by an unknown gravity influence that could not be eliminated. Consequently, this data set was excluded from further evaluation. After the surgery, the error could be attributed to a plug connection that was not sufficiently robust for intraoperative use. This connector was revised and strengthened for subsequent measurements and has worked error-free since then.

Artifacts in the force recording due to contact between the bone at the mastoid cavity or the posterior tympanotomy and the electrode holder could not be entirely avoided. In two cases 2.5% and 6.3% of the recorded force data (between the start of insertion until reaching the final depth) was overlaid by these extracochlear events, likely obscuring the intracochlear forces of interest. In the other two the patients, insertion was possible without any discernible, extracochlear bone contact.

Table 1 summarizes the key parameters of the measured forces from all four patients evaluated so far.

**Table 1:** Results of force measurement.

Patient	Duration of insertion [s]	Periods with extracochlear forces [%]	Mean force [mN]	Final force [mN]	Maximal peak force [mN]	Maximal compensated force [mN]
01	41	0.0	9.3	17.2	71.6	2.8
02	37	2.5	13.4	*	95.7	45.5
03	194	6.3	24.8	43.6	102.4	12.7
04	322	0.0	13.0	29.9	44.8	5.5

Final force is the force measured at the end of the insertion (at the point of maximal insertion depth). Maximal peak force is the highest observed force value, regardless of the time it was measured. Mean force represents the average force throughout the entire insertion process. The last column represents the maximum impact of gravitational effects included in the raw force signal, which is calculated and subtracted by the gravity compensation algorithm throughout the entire insertion process. *In patient 3, bone contact was observed at the point of full insertion; therefore, the true height of the intracochlear forces is unknown (82 mN have been measured directly before bone contact).

## 4 Discussion

The initial experiences gained intraoperatively are promising, as successful integration into the conventional workflow was possible without encountering significant obstacles. The force sensing insertion tool does not require substantial changes in the surgical workflow. In all patients, replacement of the conventional forceps with the tool was the only planned deviation from the standard protocol for cochlear implantation, which means that clamping, releasing and spatial manipulation of the EA by grasping the tool was the sole necessary difference to what the surgeons are used to do intraoperatively.

Apart from an insufficiently stable plug connection, which was reworked after its first appearance, no other problems with the measurement method itself were observed. The gravity compensation algorithm reliably removed forces that were caused by orientation changes of the tool. In some cases, the surgical video showed only small trajectory corrections (as such in the 2^nd^ patient), suggesting that the uncompensated raw data is likely still interpretable when accepting a larger measurement uncertainty. However, forces up to 45 mN have been removed in these *in vivo* trials so far, which would have otherwise disturbed the measurement. When compared with the magnitude of the forces of interest (see Table 1), this highlights that assuming a linear hand motion to omit the additional orientation sensor is not sufficient for precise insertion force measurements.

**Figure 4:**
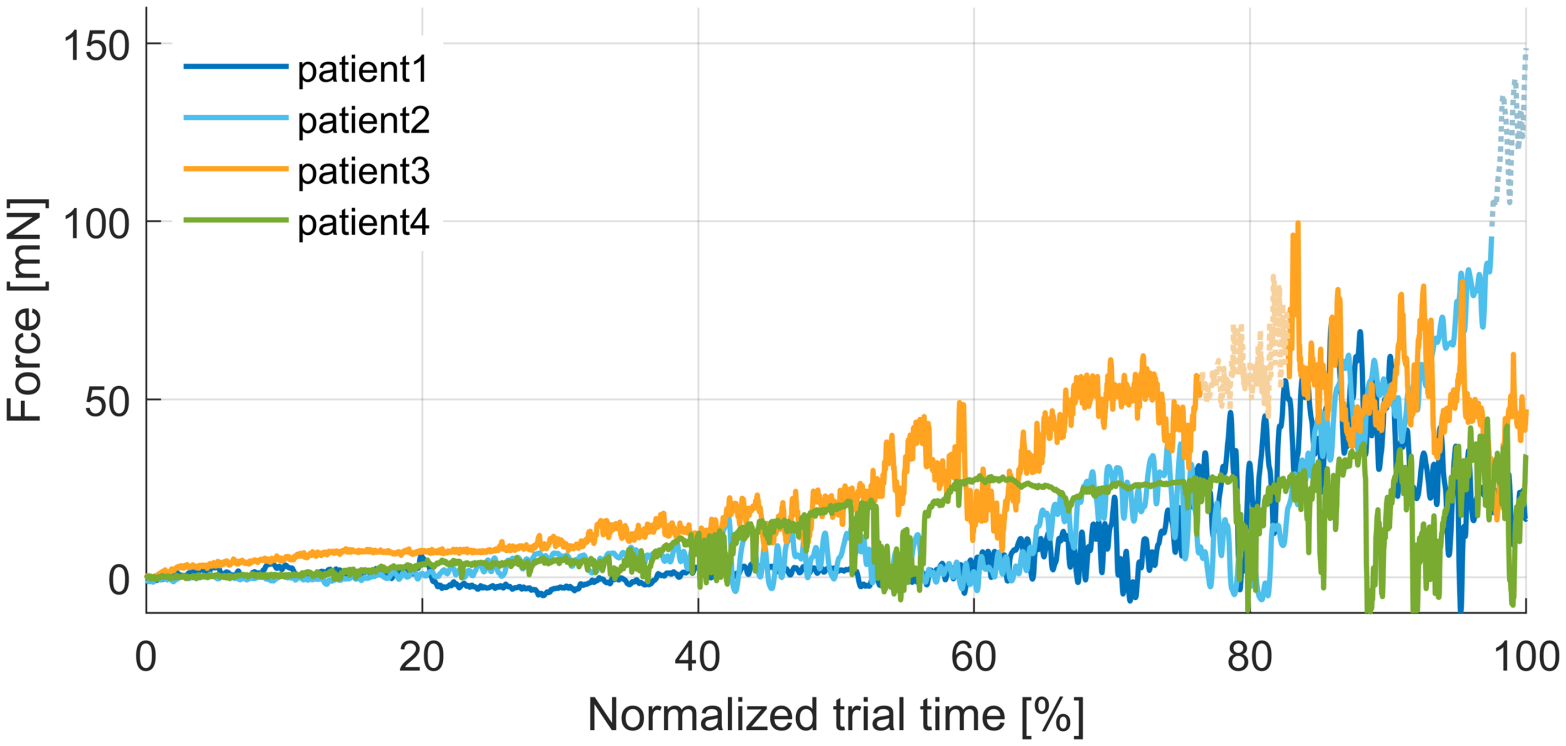
Plot of insertion forces normalized to the duration of insertion for better comparability. Periods with bone contact of the instrument tip are plotted as dashed lines and reduced color intensity in the force profiles.

The measured *in vivo* insertion forces (cleaned from artifacts due to bone contact of the electrode holder, see Figure 4) showed the expected rise with increasing insertion depth, consistent with findings from research conducted on artificial cochlear models and our previous temporal bone trials. In those *ex vivo* conditions, the values for the maximal forces ranged between 31.4 mN and 126.0 mN (*n* = 18) (19). Here, we observed maximal peak forces in the range from 44.8 mN to 102.4 mN. These peaks, which are typical for manual insertion, can appear throughout the insertion process, not necessarily toward the end. Therefore, the final forces are different, ranging from 17.2 mN to 43.6 mN in the *in vivo* data from three patients that could be evaluated so far. In a fourth patient, contact of the electrode holder with bone outside the cochlea was observed in the video. Therefore, the actual intracochlear forces might be obscured in this case, and the force value recorded at the end of this insertion is not a reliable indicator for the intracochlear events.

Temporary increases in the insertion force could mostly not be linked to a specific visible event or external observation, such as electrode buckling outside the cochlea. Based on our experience with both *ex vivo* and *in vivo* insertion trials, it appears that higher insertion forces—likely due to mechanical resistances within the cochlea—promote the occurrence of extracochlear buckling. From a mechanical point of view, buckling is an evasive movement of the electrode array when internal axial forces exceed the critical load (also known as Euler’s buckling load). Buckling of more flexible electrode arrays might therefore even shield the inner ear from high forces applied at the lateral end. Nevertheless, more extensive studies are necessary to make qualified statements about the significance of specific factors affecting insertion forces.

In general, any extracochlear contact with the tip of the tool or the EA compromises the goal of measuring intracochlear forces. This is a fundamental limitation of this method. However, touching the mastoid bone with the tool does not seem to occur as often as was initially assumed during the development. In our *ex vivo* trials, such events were discovered in only 3 out of 18 (16.6%) insertion experiments affecting between 7.6% and 17.1% of the corresponding insertions (19). In this *in vivo* study, so far, in the most severe case, only 6.3% of the entire measurement duration was affected (Table 1), demonstrating that the measurement method can be reliably used most of the time.

It is worth noting that postoperative visual detection of extracochlear contact in the videos beyond tapping the bone with the tip of the instrument is challenging due to perspective, small dimensions, and lack of spatial representation. While bone contact of the tool can be comparatively well identified as the pronounced peaks in the force facilitate visual detection in the videos, a light touch or sliding of the EA along the boundaries of the posterior tympanotomy can hardly be identified. Such contact events might slightly distort the measured forces as well and somewhat hamper the ultimate goal of only recording intracochlear forces. Even if it is possible to avoid any contact at the facial recess, there could still be contact and, consequently, friction at the boundaries of the round window opening. Although these friction forces contribute to the total insertion forces, they are not considered “intracochlear” in terms of being critical for insertion trauma. The low level of insertion forces at the beginning of the insertion (Figure 4), when the EA moves through the straight basal part of the cochlea, indicates that this component of the insertion forces is relatively small and is, therefore, likely negligible.

An ongoing consideration involves the optimal location for holding the electrode array (EA). Positioning the electrode holder directly behind the marker facilitates guiding the implant close to the round window, offering enhanced control over the entire insertion process and minimizing unsupported length, thus reducing the risk of lateral deflection (buckling). However, this placement necessitates passing the electrode holder through the facial recess, requiring sufficient width in this anatomical bottleneck and increasing the possibility of unintended contact that may interfere with the force measurement. This suggests the potential advantage of a more distal clamping of the EA. Based on our accumulated experiences both *in vivo* and *ex vivo* (19), we assume, that both approaches are viable options and their choice might depend on surgeon’s preferences as well as patient-specific anatomical factors such as the width of the facial recess.

To date, trauma prevention in clinical practice largely relies on the subjective assessments of indirect indicators for insertion trauma, such as the perceived mechanical resistance or the visual observation of electrode buckling, as manual perception of insertion forces is very limited (5). These limitations of human capabilities impede the effectiveness of achieving truly atraumatic electrode insertion. Furthermore, the inability to measure insertion forces in real time during surgery precludes thorough examination of the relationship between these forces and residual hearing preservation, as well as their impact on long-term hearing outcomes. The emergence of intracochlear force measurement methodology now offers a pathway for the in-depth exploration of these effects and causal relationships, as well as the explanatory power of insertion forces as a significant implication of electrode insertion. That again supports the development of evidence-based strategies to further improve the safety and reliability of cochlear implantation surgery with regard to residual hearing preservation.

Monitoring the insertion process to enhance the likelihood of atraumatic insertion has become a topic of growing interest in recent years. The most advanced technology in this field is intraoperative electrocochleography (ECochG) (23–25). ECochG utilizes the biofeedback of sensory cells, making it a direct measure of trauma, whereas insertion forces, by their nature, serve as an indirect measure of trauma. However, there may be distinct advantages to force measurement. The setup is simple and does not depend on the quality of the acoustic presentation or upon the amount of preoperative hearing (26). Additionally, force measurement begins immediately, while usable ECochG signals can only be obtained after reaching a certain insertion depth (26). The ECochG signal is more complex and the informative value is reliant on multiple components of the signal (9, 23, 27). On the other hand, force measurement does not allow to distinguish the place where mechanical resistance appears. Forces along the whole EA are summed up and recorded while only the forces at the tip might be relevant for insertion trauma. However, it is too early to deeply discuss and compare force measurement with ECochG. Both methods offer valuable insights into the insertion process and have the potential to aid surgeons in protecting intracochlear structures from potential damage (9).

Furthermore, fluoroscopy is considered beneficial for providing insights into the intracochlear interaction of the EA with the surrounding tissue (28). Moreover, there is consideration for combining fluoroscopy and ECochG (29). Combining fluoroscopy with force measurement in a future study would add visualization of the intracochlear movement of the EA to the recorded insertion forces, which might help to better understand the location where mechanical resistance appears and how changes in the insertion forces are related to insertion depth. Technically, a combination of all three methods is feasible, not only revealing the specific advantages of each but also facilitating complementary knowledge acquisition and conclusions.

Extending force measurement to a wider range of patients only requires adapting the electrode holder to suit the specific designs of various lateral wall EAs. Employing interchangeable electrode holders, akin to the approach used in the RobOtol system (30), offers the potential of facilitating comprehensive force measurement across different implant types and manufacturers.

Another goal for the near future is a clinical study that actually incorporates real-time intraoperative visualization using the picture-in-picture mode of the digital surgical microscope (25, 31). With respect to patient safety, this part of the whole concept was omitted in the current study, although there are no contraindications yet to pursuing that direction. Previous experiments demonstrated the high accuracy of the integrated sensor technology (18), and the participating surgeons in the ongoing study did not express concerns about potential interference with their surgical approach when this additional information is displayed.

## 5 Conclusion

Our preliminary results clearly show the feasibility of real-time measurement of insertion forces during standard cochlear implant surgery. Data acquisition was successful and surgeons quickly adapted to the minor procedural changes necessary. We hypothesize that this novel measurement tool has the potential to aid surgeons in reducing insertion trauma and ensure better residual hearing preservation. To ensure surgeons can react to force changes, a reliable and intuitive feedback method will need to be implemented. Eventually, studies involving significantly larger numbers of patients will be necessary to determine the effectiveness of force feedback in the prediction and prevention of insertion trauma and to demonstrate a clinically relevant added value for residual hearing preservation. This data can further serve as the basis for ongoing investigations into causes and effects of insertion trauma, leading to new and improved prevention strategies. Furthermore, the combination of real-time force measurement with fluoroscopy and/or ECochG has the potential to further enhance our understanding of the insertion process, providing valuable insights for complementary research.

## Declaration of generative AI and AI-assisted technologies in the writing process

Parts of the manuscript were improved by using artificial intelligence (ChatGPT 3.5) for spell checking and improving readability of single sentences. After carefully reviewing, the authors confirm that the algorithm only corrects grammar errors and typos while keeping the original meaning of the statements. The authors take full responsibility for the content of the publication.

## Data availability statement

The raw data supporting the conclusions of this article will be made available by the authors, without undue reservation.

## Ethics statement

The study involving humans was approved by local institutional review board of Hannover Medical School. The studies were conducted in accordance with the local legislation and institutional requirements. The participants provided their written informed consent to participate in this study.

## Author contributions

TR: Conceptualization, Formal analysis, Funding acquisition, Investigation, Methodology, Project administration, Resources, Supervision, Validation, Visualization, Writing – original draft, Writing – review & editing. GB-R: Data curation, Formal analysis, Investigation, Methodology, Software, Validation, Visualization, Writing – original draft, Writing – review & editing. VS: Data curation, Investigation, Methodology, Software, Writing – review & editing. JC: Investigation, Writing – review & editing. EA: Resources, Writing – review & editing. CB: Methodology, Writing – review & editing. TL: Conceptualization, Funding acquisition, Investigation, Supervision, Writing – review & editing. RS: Investigation, Resources, Writing – review & editing.
